# ADAPTING STEADI TOOLKIT FOR LONG-TERM CARE: A DELPHI STUDY

**DOI:** 10.1093/geroni/igae098.3360

**Published:** 2024-12-31

**Authors:** Hilaire Thompson, Elizabeth Phelan, Ellen McGough, Kushang Patel

**Affiliations:** University of Washington, Seattle, Washington, United States; University of Washington, Seattle, Washington, United States; University of Rhode Island, Kingston, Rhode Island, United States; University of Washington, Seattle, Washington, United States

## Abstract

The prevalence of falls for older adults in long-term care facilities (LTCF) is estimated to be 50-60%. However, fall prevention programs, such as the CDC’s STEADI, have not been validated for use in LTCF. Lacking are screening, assessment, and decision-support toolkits to be used with older adults residing in LTCF. This project seeks to address this gap by developing a refined toolkit for use in LTCF. As a first step, an initial list of adaptations to the STEADI toolkit were identified following four separate scoping reviews. Interventions were categorized in six areas: Assessment, Education/Training, Facility, Medication Management, Resident Lifestyle, and Referral. Subject matter experts from medicine, nursing, physical therapy, and long-term care administration from across the US were invited to provide feedback on the initial draft of toolkit interventions. Interviews (n=9) were transcribed and recommendations for changes to assessment and management strategies were identified. Following these revisions, we recruited individuals working in LTCFs to participate in two rounds of surveys using a modified Delphi technique. Respondents (n=51) were asked to rate 69 interventions as either not at all helpful, possibly helpful, helpful or extremely helpful. We established consensus as =/>60% of individuals agreeing that the intervention was “extremely helpful.” In round one, 37 interventions reached consensus for inclusion (Assess: 16; Education/Training: 4; Facility: 8; Medication Management: 2; Resident Lifestyle: 4; Referral: 3). In round two, three additional items reached consensus for inclusion. The toolkit is now being tested for feasibility, acceptability and impact on falls and fall-related injuries.

